# Poly[bis­(μ_7_-3-sulfonato-l-alaninato)sodiumzinc]

**DOI:** 10.1107/S160053681202394X

**Published:** 2012-05-31

**Authors:** Xiao-lin Li, Jing Yu, Li Liu

**Affiliations:** a Physics and Chemistry Department, Jiangxi College of Traditional Chinese Medicine, Fuzhou, Jiangxi 344000, People’s Republic of China

## Abstract

The hydro­thermal reaction of Zn(CH_3_COO)_2_, NaOH and l-cysteic acid produced the title compound, [Na_2_Zn(C_3_H_5_NO_5_S)_2_]_*n*_. The Zn^II^ cation is situated on an inversion centre and is in a distorted octa­hedral environment, being chelated by two deprotoned l-cysteic acid ligands through two amino N atoms and two carb­oxy­lic O atoms, with the two axial positions occupied by two carb­oxy­lic O atoms from two other l-cysteic acid ligands. Each l-cysteic acid ligand bridges five Na^I^ ions *via* its sulfonate group and two Zn^II^ ions *via* its carboxyl group, forming a three-dimensional framework. Weak N—H⋯O hydrogen bonding is observed in the crystal structure.

## Related literature

For general background to l-cysteic acid complexes, see: Li *et al.* (2009 ▶, 2011*a* ▶,*b* ▶); Huang *et al.* (2009 ▶). 
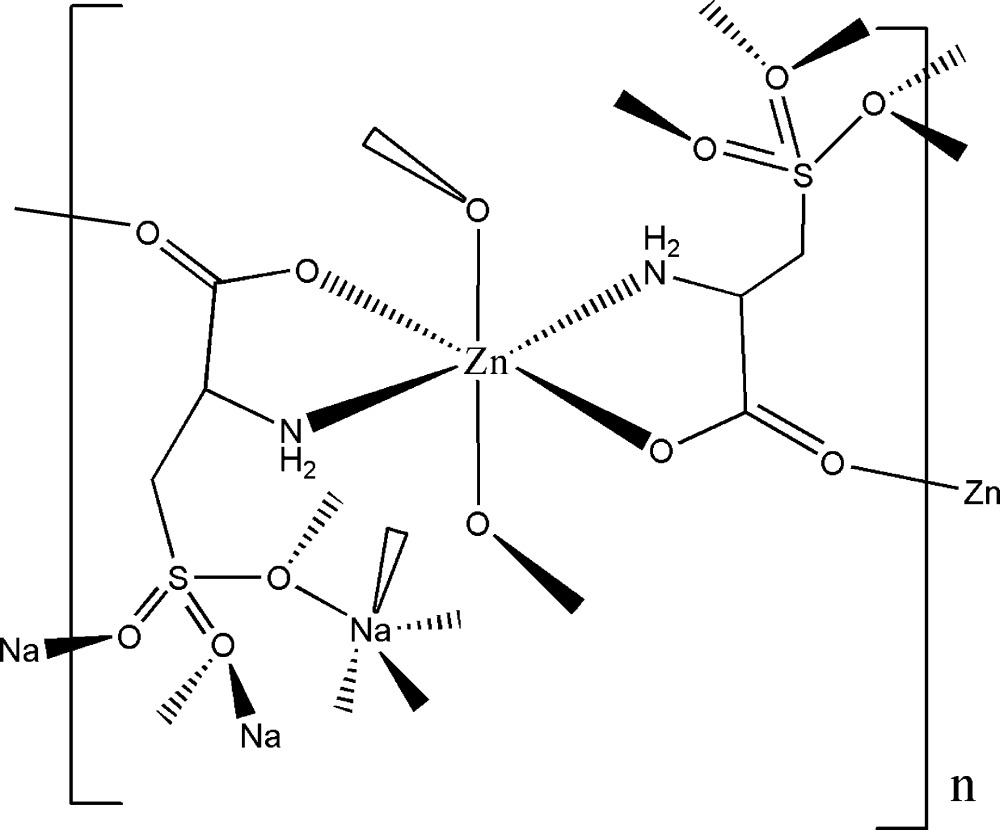


## Experimental

### 

#### Crystal data

[Na_2_Zn(C_3_H_5_NO_5_S)_2_]
*M* *_r_* = 445.67
Monoclinic, 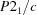

*a* = 13.2432 (6) Å
*b* = 6.1574 (2) Å
*c* = 8.5959 (3) Å
β = 98.155 (2)°
*V* = 693.85 (5) Å^3^
*Z* = 2
Mo *K*α radiation
μ = 2.19 mm^−1^
*T* = 296 K
0.22 × 0.18 × 0.12 mm

#### Data collection

Bruker SMART CCD area-detector diffractometer
Absorption correction: multi-scan (*SADABS*; Bruker, 1999 ▶) *T*_min_ = 0.629, *T*_max_ = 0.769
5309 measured reflections
1293 independent reflections
1280 reflections with *I* > 2σ(*I*)
*R*_int_ = 0.018

#### Refinement

*R*[*F* ^2^ > 2σ(*F* ^2^)] = 0.027
*wR*(*F* ^2^) = 0.075
*S* = 0.97
1293 reflections
106 parameters
H-atom parameters constrained
Δρ_max_ = 0.40 e Å^−3^
Δρ_min_ = −0.45 e Å^−3^

### 

Data collection: *SMART* (Bruker, 1999 ▶); cell refinement: *SAINT* (Bruker, 1999 ▶); data reduction: *SAINT*; program(s) used to solve structure: *SHELXS97* (Sheldrick, 2008 ▶); program(s) used to refine structure: *SHELXL97* (Sheldrick, 2008 ▶); molecular graphics: *SHELXTL* (Sheldrick, 2008 ▶); software used to prepare material for publication: *SHELXTL*.

## Supplementary Material

Crystal structure: contains datablock(s) I, global. DOI: 10.1107/S160053681202394X/zj2077sup1.cif

Structure factors: contains datablock(s) I. DOI: 10.1107/S160053681202394X/zj2077Isup2.hkl

Additional supplementary materials:  crystallographic information; 3D view; checkCIF report

## Figures and Tables

**Table 1:** Selected bond lengths (Å)

Zn1—O4^i^	2.0588 (18)
Zn1—O4	2.0588 (18)
Zn1—N1	2.116 (2)
Zn1—N1^i^	2.116 (2)
Zn1—O5^ii^	2.195 (2)
Zn1—O5^iii^	2.195 (2)
Na1—O2^iv^	2.309 (2)
Na1—O1	2.347 (2)
Na1—O1^v^	2.442 (2)
Na1—O3^vi^	2.386 (2)
Na1—O2^vii^	2.426 (3)

Symmetry codes: (i) 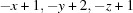; (ii) 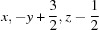; (iii) 
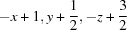
; (iv) 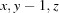; (v) 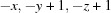; (vi) 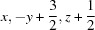; (vii) 
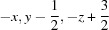
.

**Table 2:** Hydrogen-bond geometry (Å, °)

*D*—H⋯*A*	*D*—H	H⋯*A*	*D*⋯*A*	*D*—H⋯*A*
N1—H1*B*⋯O3	0.90	2.28	3.030 (3)	141
N1—H1*A*⋯O4^iii^	0.90	2.28	2.860 (3)	122

Symmetry code: (iii) 
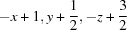
.

**Fig. 1.**
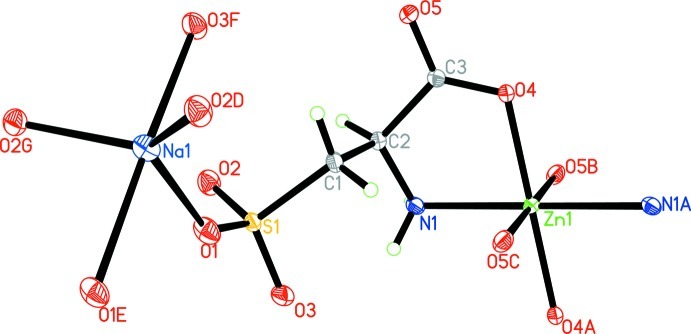
The molecular structure of the title compound, with 30% probability displacement ellipsoids and the atom-numbering scheme. [Symmetry code: (A) 1 - x, 2 - y, 1 - z; (B) 1 - x, y + 1/2, 3/2 - z; (C) x, -y + 3/2, z - 1/2; (D) x, y - 1, z; (E) -x, -y + 1, 1 - z;(F)x, 3/2 - y, 1/2 + z;(G)-x, -1/2 + y, 3/2 - z.]

**Fig. 2.**
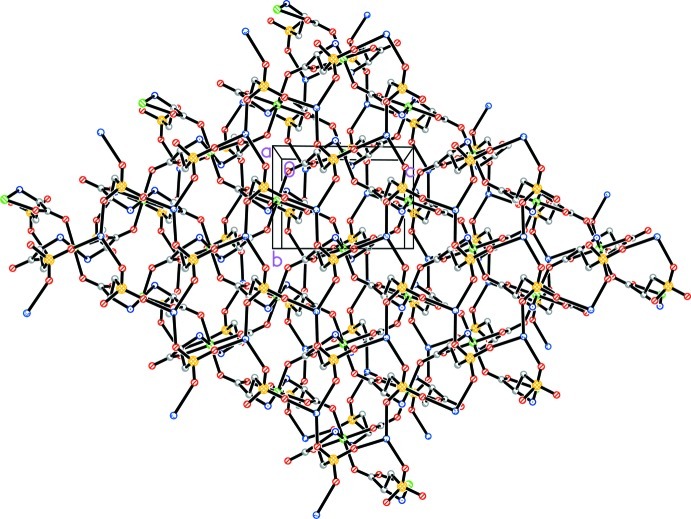
The crystal packing of the title compound viewed down the a axis.Hydrogen atoms have be omitted for clarity.

## supplementary crystallographic information

### Comment

*L*-cysteic acid, an amino acid containing containing both sulfur and carboxyl, is indispensable to human beings with important physiologic functions. Recently,Jiang reported many compounds containing *L*-cysteic acid [Li *et al.* (2009, 2011*a*,*b*);Huang *et al.* (2009)]. *L*-cysteic acid, as a multidentate ligand with six coordination sites might be utilized as a versatile linker in the construction of interesting multidimensional complexes with the capability of participating in hydrogen bonding with multiproton acceptor or donor sites, which is a candidate for construction of multidimensional complexes. Herein, we present a new coordination polymer [ZnNa(C_3_H_5_NO_5_S)_2_]n (Scheme 1, Fig. 1). Each Zn(II) ion is six-coordinated to four oxygen atoms (O4, O4A, O5B, O5C), which belonging to four different *L*-cysteic acid ligands, two amino nitrogen atom (N1, N1A) from different ligands to give a distorted octahedron geometries. Each sulfonate group of the taurinate ligand takes part in the formation of a hydrogen bond (Table 2) with the amino group of a neighbouring ligand in the complex. A notable feature of the title complex lies in the coordination modes of the sulfonate group. The most common coordination modes are monodentate and µ_2_ or µ_3_-bridging, while µ_5_-bridging is very rare. The Na atom is surrounded by five O atoms from different ligands, The title complex forms a three-dimensional structure (Fig. 2) through the Na···O linkage. The Na···O distances are in the range 2.309 (2)–2.442 (2) Å, suggesting weak electrostatic interactions.

### Experimental

A mixture of Zn(CH_3_COO)_2_ (0.5 mmol, 92.5 mg), *L*-cysteic acid (1.0 mmol 169 mg), NaOH (2.0 mmol, 80 mg) and anhydrous methanol (15.0 ml) was placed in a Teflon-lined stainless steel vessel, and heated directly to 115 °C. After keeping at 115 °C for 5 days, it was cooled to room temperature at a rate for 10 °C/h. block colorless crystals of the complex were obtained.

### Refinement

H atoms were positioned geometrically (C–H = 0.97 Å and N–H = 0.90 Å) and included in the refinement in the riding model approximation, with *U*_iso_(H) = 1.2*U*_eq_(carrier atom).

### Crystal data

**Table:**

[Na_2_Zn(C_3_H_5_NO_5_S)_2_]	*Z* = 2
*M**_r_* = 445.67	*F*(000) = 448
Monoclinic, *P*2_1_/*c*	*D*_x_ = 2.133 Mg m^−^^3^
Hall symbol: -P 2ybc	Mo *K*α radiation, λ = 0.71073 Å
*a* = 13.2432 (6) Å	µ = 2.19 mm^−^^1^
*b* = 6.1574 (2) Å	*T* = 296 K
*c* = 8.5959 (3) Å	Block, colorless
β = 98.155 (2)°	0.22 × 0.18 × 0.12 mm
*V* = 693.85 (5) Å^3^	

### Data collection

**Table:**

Bruker SMART CCD area-detector diffractometer	1293 independent reflections
Radiation source: fine-focus sealed tube	1280 reflections with *I* > 2σ(*I*)
Graphite monochromator	*R*_int_ = 0.018
phi and ω scans	θ_max_ = 25.5°, θ_min_ = 3.1°
Absorption correction: multi-scan (*SADABS*; Bruker, 1999)	*h* = −16→12
*T*_min_ = 0.629, *T*_max_ = 0.769	*k* = −7→7
5309 measured reflections	*l* = −10→10

### Refinement

**Table:**

Refinement on *F*^2^	Primary atom site location: structure-invariant direct methods
Least-squares matrix: full	Secondary atom site location: difference Fourier map
*R*[*F*^2^ > 2σ(*F*^2^)] = 0.027	Hydrogen site location: inferred from neighbouring sites
*wR*(*F*^2^) = 0.075	H-atom parameters constrained
*S* = 0.97	*w* = 1/[σ^2^(*F*_o_^2^) + (0.0389*P*)^2^ + 1.860*P*] where *P* = (*F*_o_^2^ + 2*F*_c_^2^)/3
1293 reflections	(Δ/σ)_max_ < 0.001
106 parameters	Δρ_max_ = 0.40 e Å^−^^3^
0 restraints	Δρ_min_ = −0.45 e Å^−^^3^

### Special details

**Table:**

Geometry. All e.s.d.'s (except the e.s.d. in the dihedral angle between two l.s. planes) are estimated using the full covariance matrix. The cell e.s.d.'s are taken into account individually in the estimation of e.s.d.'s in distances, angles and torsion angles; correlations between e.s.d.'s in cell parameters are only used when they are defined by crystal symmetry. An approximate (isotropic) treatment of cell e.s.d.'s is used for estimating e.s.d.'s involving l.s. planes.
Refinement. Refinement of *F*^2^ against ALL reflections. The weighted *R*-factor *wR* and goodness of fit *S* are based on *F*^2^, conventional *R*-factors *R* are based on *F*, with *F* set to zero for negative *F*^2^. The threshold expression of *F*^2^ > σ(*F*^2^) is used only for calculating *R*-factors(gt) *etc*. and is not relevant to the choice of reflections for refinement. *R*-factors based on *F*^2^ are statistically about twice as large as those based on *F*, and *R*- factors based on ALL data will be even larger.

### Fractional atomic coordinates and isotropic or equivalent isotropic displacement parameters (Å^2^)

**Table:**

	*x*	*y*	*z*	*U*_iso_*/*U*_eq_	
Zn1	0.5000	1.0000	0.5000	0.02019 (15)	
Na1	0.03657 (9)	0.38146 (19)	0.69254 (13)	0.0282 (3)	
S1	0.13597 (5)	0.88954 (11)	0.57241 (8)	0.01901 (18)	
O1	0.07432 (16)	0.6925 (3)	0.5538 (3)	0.0318 (5)	
O2	0.10261 (16)	1.0349 (4)	0.6877 (3)	0.0312 (5)	
O3	0.14300 (16)	0.9951 (4)	0.4229 (3)	0.0323 (5)	
O4	0.51364 (13)	0.8902 (3)	0.7282 (2)	0.0206 (4)	
O5	0.42196 (14)	0.8016 (3)	0.9167 (2)	0.0245 (4)	
N1	0.35961 (16)	1.1125 (4)	0.5602 (3)	0.0195 (5)	
H1A	0.3642	1.2549	0.5836	0.023*	
H1B	0.3098	1.0943	0.4784	0.023*	
C1	0.26104 (19)	0.8023 (5)	0.6488 (3)	0.0201 (5)	
H1C	0.2573	0.7106	0.7395	0.024*	
H1D	0.2877	0.7148	0.5700	0.024*	
C2	0.3354 (2)	0.9888 (4)	0.6971 (3)	0.0194 (6)	
H2	0.3050	1.0872	0.7672	0.023*	
C3	0.4327 (2)	0.8863 (4)	0.7882 (3)	0.0183 (5)	

### Atomic displacement parameters (Å^2^)

**Table:**

	*U*^11^	*U*^22^	*U*^33^	*U*^12^	*U*^13^	*U*^23^
Zn1	0.0172 (2)	0.0295 (3)	0.0144 (2)	0.00005 (17)	0.00421 (16)	0.00207 (17)
Na1	0.0300 (6)	0.0232 (6)	0.0301 (6)	0.0004 (5)	0.0000 (5)	−0.0026 (5)
S1	0.0132 (3)	0.0225 (3)	0.0208 (3)	−0.0017 (2)	0.0004 (2)	0.0025 (3)
O1	0.0279 (11)	0.0259 (11)	0.0389 (12)	−0.0079 (9)	−0.0044 (9)	0.0025 (9)
O2	0.0265 (11)	0.0326 (11)	0.0344 (12)	0.0052 (9)	0.0041 (9)	−0.0055 (9)
O3	0.0243 (11)	0.0450 (14)	0.0265 (11)	−0.0006 (9)	−0.0005 (9)	0.0129 (9)
O4	0.0135 (9)	0.0303 (11)	0.0184 (9)	0.0022 (8)	0.0034 (7)	0.0047 (8)
O5	0.0216 (9)	0.0343 (11)	0.0188 (9)	0.0063 (9)	0.0063 (8)	0.0069 (8)
N1	0.0165 (11)	0.0221 (11)	0.0190 (11)	−0.0022 (9)	−0.0003 (8)	0.0044 (9)
C1	0.0149 (12)	0.0230 (13)	0.0225 (13)	0.0006 (11)	0.0030 (10)	0.0023 (11)
C2	0.0146 (12)	0.0248 (14)	0.0188 (13)	0.0008 (10)	0.0027 (10)	0.0028 (10)
C3	0.0171 (12)	0.0205 (13)	0.0170 (12)	0.0012 (10)	0.0013 (10)	−0.0003 (10)

### Geometric parameters (Å, º)

**Table:**

Zn1—O4^i^	2.0588 (18)	O1—Na1^v^	2.442 (2)
Zn1—O4	2.0588 (18)	O2—Na1^viii^	2.309 (2)
Zn1—N1	2.116 (2)	O2—Na1^ix^	2.426 (3)
Zn1—N1^i^	2.116 (2)	O3—Na1^ii^	2.386 (2)
Zn1—O5^ii^	2.195 (2)	O4—C3	1.254 (3)
Zn1—O5^iii^	2.195 (2)	O5—C3	1.248 (3)
Na1—O2^iv^	2.309 (2)	O5—Zn1^x^	2.195 (2)
Na1—O1	2.347 (2)	N1—C2	1.474 (3)
Na1—O1^v^	2.442 (2)	N1—H1A	0.9000
Na1—O3^vi^	2.386 (2)	N1—H1B	0.9000
Na1—O2^vii^	2.426 (3)	C1—C2	1.531 (4)
S1—O2	1.450 (2)	C1—H1C	0.9700
S1—O3	1.455 (2)	C1—H1D	0.9700
S1—O1	1.458 (2)	C2—C3	1.545 (4)
S1—C1	1.776 (3)	C2—H2	0.9800
			
O4^i^—Zn1—O4	180.0	Na1—O1—Na1^v^	98.23 (8)
O4^i^—Zn1—N1	99.45 (8)	S1—O2—Na1^viii^	137.36 (15)
O4—Zn1—N1	80.55 (8)	S1—O2—Na1^ix^	111.91 (13)
O4^i^—Zn1—N1^i^	80.55 (8)	Na1^viii^—O2—Na1^ix^	92.28 (8)
O4—Zn1—N1^i^	99.45 (8)	S1—O3—Na1^ii^	140.46 (14)
N1—Zn1—N1^i^	180.0	C3—O4—Zn1	115.68 (16)
O4^i^—Zn1—O5^ii^	89.62 (8)	C3—O5—Zn1^x^	122.34 (17)
O4—Zn1—O5^ii^	90.38 (8)	C2—N1—Zn1	108.92 (16)
N1—Zn1—O5^ii^	88.13 (8)	C2—N1—H1A	109.9
N1^i^—Zn1—O5^ii^	91.87 (8)	Zn1—N1—H1A	109.9
O4^i^—Zn1—O5^iii^	90.38 (8)	C2—N1—H1B	109.9
O4—Zn1—O5^iii^	89.62 (8)	Zn1—N1—H1B	109.9
N1—Zn1—O5^iii^	91.87 (8)	H1A—N1—H1B	108.3
N1^i^—Zn1—O5^iii^	88.13 (8)	C2—C1—S1	113.79 (19)
O5^ii^—Zn1—O5^iii^	180.0	C2—C1—H1C	108.8
O2^iv^—Na1—O1	129.53 (9)	S1—C1—H1C	108.8
O2^iv^—Na1—O3^vi^	97.40 (9)	C2—C1—H1D	108.8
O1—Na1—O3^vi^	91.05 (9)	S1—C1—H1D	108.8
O2^iv^—Na1—O2^vii^	132.84 (9)	H1C—C1—H1D	107.7
O1—Na1—O2^vii^	97.31 (9)	N1—C2—C1	112.0 (2)
O3^vi^—Na1—O2^vii^	85.34 (8)	N1—C2—C3	110.8 (2)
O2—S1—O3	113.08 (14)	C1—C2—C3	106.8 (2)
O2—S1—O1	111.61 (13)	N1—C2—H2	109.0
O3—S1—O1	112.31 (13)	C1—C2—H2	109.0
O2—S1—C1	107.02 (13)	C3—C2—H2	109.0
O3—S1—C1	106.76 (13)	O5—C3—O4	125.7 (2)
O1—S1—C1	105.50 (13)	O5—C3—C2	115.4 (2)
S1—O1—Na1	141.19 (13)	O4—C3—C2	119.0 (2)
S1—O1—Na1^v^	120.57 (13)		

Symmetry codes: (i) −*x*+1, −*y*+2, −*z*+1; (ii) *x*, −*y*+3/2, *z*−1/2; (iii) −*x*+1, *y*+1/2, −*z*+3/2; (iv) *x*, *y*−1, *z*; (v) −*x*, −*y*+1, −*z*+1; (vi) *x*, −*y*+3/2, *z*+1/2; (vii) −*x*, *y*−1/2, −*z*+3/2; (viii) *x*, *y*+1, *z*; (ix) −*x*, *y*+1/2, −*z*+3/2; (x) −*x*+1, *y*−1/2, −*z*+3/2.

### Hydrogen-bond geometry (Å, º)

**Table:**

*D*—H···*A*	*D*—H	H···*A*	*D*···*A*	*D*—H···*A*
N1—H1*B*···O3	0.90	2.28	3.030 (3)	141
N1—H1*A*···O4^iii^	0.90	2.28	2.860 (3)	122

Symmetry code: (iii) −*x*+1, *y*+1/2, −*z*+3/2.
